# Novel Strategies for the Generation of Neuronal Diversity: Lessons From the Fly Visual System

**DOI:** 10.3389/fnmol.2019.00140

**Published:** 2019-05-31

**Authors:** Esteban G. Contreras, Jimena Sierralta, Carlos Oliva

**Affiliations:** ^1^Department of Neuroscience and Biomedical Neuroscience Institute, Faculty of Medicine, Universidad de Chile, Santiago, Chile; ^2^Department of Cellular and Molecular Biology, Faculty of Biological Sciences, Pontificia Universidad Católica de Chile, Santiago, Chile

**Keywords:** central nervous system, neurogenesis, *Drosophila melanogaster*, visual system, optic lobe, lobula plate development, inner proliferation center

## Abstract

Among all organs of an adult animal, the central nervous system stands out because of its vast complexity and morphological diversity. During early development, the entire central nervous system develops from an apparently homogenous group of progenitors that differentiate into all neural cell types. Therefore, understanding the molecular and genetic mechanisms that give rise to the cellular and anatomical diversity of the brain is a key goal of the developmental neurobiology field. With this aim in mind, the development of the central nervous system of model organisms has been extensively studied. From more than a century, the mechanisms of neurogenesis have been studied in the fruit fly *Drosophila melanogaster*. The visual system comprises one of the major structures of the *Drosophila* brain. The visual information is collected by the eye-retina photoreceptors and then processed by the four optic lobe ganglia: the lamina, medulla, lobula and lobula plate. The molecular mechanisms that originate neuronal diversity in the optic lobe have been unveiled in the past decade. In this article, we describe the early development and differentiation of the lobula plate ganglion, from the formation of the optic placode and the inner proliferation center to the specification of motion detection neurons. We focused specifically on how the precise combination of signaling pathways and cell-specific transcription factors patterns the pool of neural stem cells that generates the different neurons of the motion detection system.

## Introduction

The vast morphological and cellular diversity displayed by the anatomy of the nervous system has fascinated neuroscientists for centuries. Different structures of the nervous system are responsible for specific tasks, and this diversity is not only reflected in distinct anatomical features, but also in specific developmental origins. Therefore, it is reasonable to believe that different cellular and molecular mechanisms govern the development of specific structures of the nervous system to attain this anatomical diversity. An example of this variety of strategies can be found in the early development of the vertebrate brain and spinal cord that originate from the neural tube, while the development of the eyes involves the interaction of the optic vesicles with the head ectoderm, which induces the formation of the lens placodes. Thus, a major challenge of the developmental neurobiology field is to understand how this diversity originates at the molecular level.

To accomplish this goal, scientists have studied the development of the nervous system using different models, including human organoids, primates, rodents and fish, but also invertebrate model organisms such as *Caenorhabditis elegans* and *Drosophila melanogaster*. Despite the evolutionary distance, neurogenesis in *Drosophila* shares fundamental cellular and molecular mechanisms with vertebrates. Therefore, by understanding this simple system, we can learn about the molecular control of vertebrate neural development.

**FIGURE 1:**
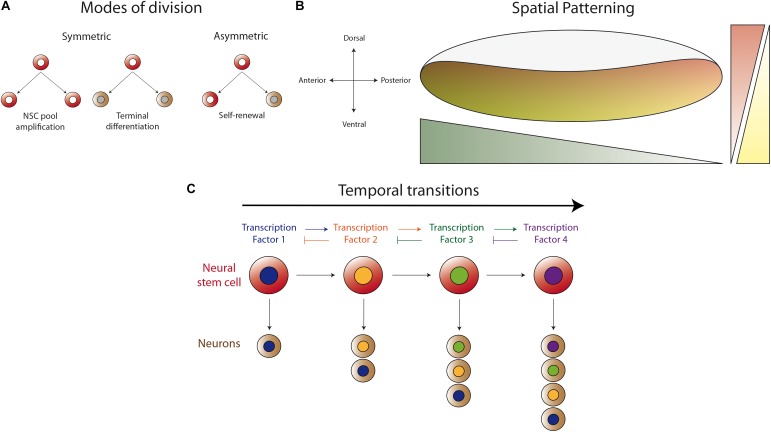
Basic mechanisms for generating neuronal diversity. **(A)** Different modes of neural stem cell division (NSC). Neural stem cells can divide in a symmetric manner to amplify their pool or to generate two progenies (neurons or glial cells) that enter the differentiation program. Asymmetric division self-renews the neural stem cell and generates a differentiating progeny, maintaining the total number of neural stem cells during neurogenesis. **(B)** Spatial patterning of the neurogenic region in the antero-posterior and dorso-ventral axes by the differential expression of transcription factors or the activity of morphogen gradients. Thus, the neurogenic tissue is patterned in the different axes to generate different types of neurons according to their spatial position. **(C)** Temporal regulation of progeny formation by a cascade of transcription factors (TF). The progeny generated in the first temporal window, defined by TF1, acquired a determined identity. TF1 activates the expression of TF2, which represses the expression of TF1, allowing the progression into the second temporal window. Thus, the neural stem cell generates a second type of progeny until progression into the next temporal window defined by another TF. This mechanism permits each neural stem cell to generate different types of neurons at different stages of animal development.

After several decades studying the development of distinct regions of the *Drosophila* central nervous system, it has become clear that there are common strategies used to produce the vast diversity of neuronal types found in this system. These strategies include: the different modes of neural stem cell division (symmetric and asymmetric, Figure 1A), the spatial patterning of neurogenic tissue across the antero-posterior and dorso-ventral axes (Figure 1B), and the generation of distinct progenies from the same neural stem cell during several temporal windows defined by the expression of different transcription factors (Figure 1C). These mechanisms for achieving diversity were first described in the embryonic ventral nerve cord, where the first temporal series of transcription factors was uncovered (Kohwi and Doe, 2013). Strikingly, although it has been shown that this basic program is present in different regions of the central nervous system, the actual sequence of transcriptional regulators is different. Currently, it is well stablished that these programs also apply during the development of the optic lobe, but with modifications that provide uniqueness to the developing visual system.

In this review, we describe the development of the *Drosophila* optic lobe and analyze recent findings on the mechanisms of neurogenesis in the visual system. We discuss the development of the optic lobe from optic placode formation to late neuronal differentiation with a special focus on early development of the lobula plate motion detection neurons, including neuronal fate specification, neuropil compartmentalization and neuronal network wiring. These topics are less covered in previous articles, but recent findings highlight novel mechanisms of neurogenesis that could be conserved throughout evolution.

## The *Drosophila* Visual System: a Model for Understanding the Mechanisms of Neurogenesis

The interaction of animals with the environment requires the sensing and processing of information in an efficient and reliable manner in order to trigger the most appropriate response. For visual stimuli, vertebrates and insects share, at the anatomical level, a common strategy for processing this information. Over a century ago, Ramon y Cajal and Sánchez described the anatomy of the insect visual system in great detail, remarking the morphological parallelism between invertebrates and vertebrates (Ramón y Cajal and Sánchez, 1915). Nowadays, research on *Drosophila melanogaster* has complemented these anatomical studies with functional and genetic evidence describing common molecular mechanisms during the neurogenesis of the visual system (Sanes and Zipursky, 2010) (Check Table 1 for a summary of genes that control the development of the *Drosophila* visual system).

**Table 1:** Summary of genes that regulate optic lobe development in *Drosophila melanogaster.*

Gene name	Symbol	Molecular function	Domain/Family	Human Ortholog gene
Abdominal-B	Abd-B	Transcription factor	Hox-like Homeobox domain	HOXA11, HOXD11
Abnormal chemosensory jump 6	Acj6	Transcription factor	POU homeobox domain	POU4F1, POU4F2, POU4F3
Asense	Ase	Transcription factor	bHLH domain	ASCL1
Atonal	Ato	Transcription factor	bHLH domain	ATOH1, ATOH7
Brain tumor	Brat	RNA binding	B-box-type zinc finger domain	TRIM2, TRIM3, and TRIM32
Brinker	Brk	Transcription factor	DNA binding domain	–
Dachshund	Dac	Transcription factor	DNA binding domain	DACH1, DACH2
Deadpan	Dpn	Transcription factor	bHLH domain	HES1
Decapentaplegic	Dpp	Secreted ligand	TGF-β signaling	BMP family
Dichaete	D	Transcription factor	HMG domain	SOX B2 family
Distal-less	Dll	Transcription factor	NK-like homeobox	DLX1, DLX6
Eph receptor tyrosine kinase	Eph	Membrane receptor	Receptor tyrosine kinase	EPH family
Ephrin	Ephrin	Secreted ligand	Ephrin receptor binding	EFNA and EFNB family
Epidermal growth factor receptor	EGFR	Membrane receptor	Receptor tyrosine kinase, EGF signaling	ERBB family
Escargot	Esg	Transcription factor	C2H2 zinc finger domain	SNAI2
Eyeless	Ey	Transcription factor	Paired Homeobox domain	PAX6
Eyes absent	Eya	Transcription cofactor	Protein Phosphatase	EYA family
Fasciclin 3	Fas3	Cell adhesion	Immunoglobulin-like domain	NCAMs
Frazzled	Fra	Membrane receptor	Immunoglobulin-like domain, Netrin signaling	NEO1, DCC
Hedgehog	Hh	Secreted ligand	Hedgehog signaling	SHH, DHH and IHH
Homothorax	Hth	Transcription factor	Homeobox domain	MEIS1
Klumpfuss	Klu	Transcription factor	C2H2 zinc finger domain	WT1
Lethal of scute	L’sc	Transcription factor	bHLH domain	ASCL1
Miranda	Mira	Scaffold protein	Protein binding	–
Netrin-A	NetA	Secreted ligand	Netrin domain	Netrin family
Netrin-B	NetB	Secreted ligand	Netrin domain	Netrin family
Notch	N	Membrane receptor	Notch signaling	NOTCH family
Numb	Numb	Membrane associated protein	Numb domain, Notch signaling	NUMBL, NUMB
Polycomblike	Pcl	Chromatin binding	PHD-type zinc finger domain	PHF1, MTF2 and PHF19
Pointed	Pnt	Transcription factor	Ets domain, EGF signaling	ETS1 and ETS2
Prospero	Pros	Transcription factor	Homeo-prospero domain	PROX1 and PROX2
Optix	Optix	Transcription factor	Homeobox domain	SIX3 and SIX6
Optomotor-blind	Omb	Transcription factor	T-box	TBX2 and TBX3
Retinal homeobox	Rx	Transcription factor	Paired-like homeobox domain	RAX and RAX2
Roundabout 1	Robo1	Membrane receptor	Immunoglobulin-like domain	ROBO family
Roundabout 2	Robo2	Membrane receptor	Immunoglobulin-like domain	ROBO family
Roundabout 3	Robo3	Membrane receptor	Immunoglobulin-like domain	ROBO family
Sine oculis	So	Transcription factor	Homeobox domain	SIX1 and SIX2
Slit	Sli	Secreted ligand	Robo signaling	SLIT1, SLIT2 and SLIT3
Sloppy paired 1/2	Slp1/2	Transcription factor	Fork head domain	FOXG1
SoxD/Sox102F	SoxD	Transcription factor	HMG domain	SOX D family
SoxNeuro	SoxN	Transcription factor	HMG domain	SOXB1 family
Tailless	Tll	Transcription factor	Nuclear hormone receptor	TLX/NR2E1
Twin of eyeless	Toy	Transcription factor	Paired homeobox domain	PAX6
Unc-5	Unc-5	Membrane receptor	Netrin signaling	UNC5A-D
Visual system homeobox 1	Vsx1	Transcription factor	Paired-like homeobox	VSX1 and VSX2
Wingless	Wg	Secreted ligand	Wnt/Wg signaling	WNT family

**Table 2:** List of abbreviations.

Abbreviation	Cell type or structure
d-IPC	Distal inner proliferation center
EMT	Epithelial-mesenchymal transition
EONs	Embryonic optic lobe neuroblasts
GMC	Ganglion mother cell
GPC	Glial precursor cells
IPC	Inner proliferation center
NB	Neuroblast
NE cells	Neuroepithelial cells
NSC	Neural stem cell
OPC	Outer proliferation center
p-IPC	Proximal inner proliferation center
s-IPC	Surface inner proliferation center

**FIGURE 2:**
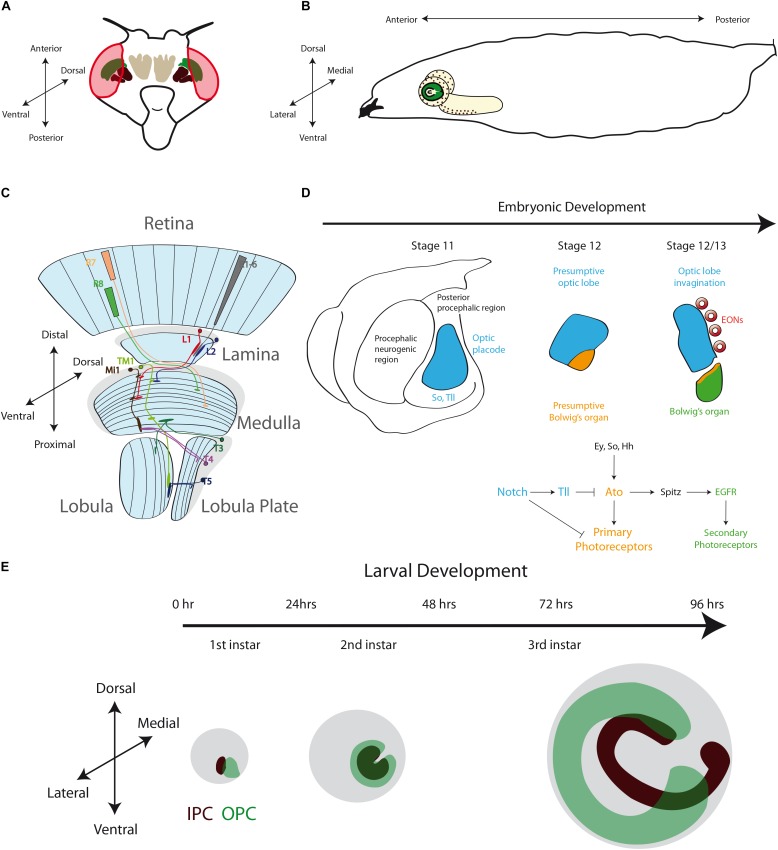
The Development of the *Drosophila* Optic Lobe. **(A)** Schematic representation of an adult head and **(B)** a larva showing the brain with its respective axes in the context of the whole animal. **(C)** The adult visual system (horizontal view) is composed by the eye retina and the optic lobe neuropils: Lamina, Medulla, Lobula, and Lobula plate. L, lamina monopolar cells; Lp, lobula plate layer; Mi, medulla intrinsic neuron; R, photoreceptor neurons (retinula cells); T, T-cells; Tm, transmedullary neurons. **(D)** Embryonic development of the optic placode. The optic placode originates at the posterior procephalic region at stage 11, and then segregates into the presumptive optic lobe and the presumptive Bolwig’s organ. At stages 12/13, the optic lobe placode invaginates and generates embryonic optic lobe neuroblasts (EONs). **(E)** During larval development, the optic lobe divides into inner and outer proliferation centers (IPC, expressing Fasciclin 3, in red, and OPC in green). Check Table 2 for a list of abbreviations.

The anatomy and neuronal circuits of the adult *Drosophila* visual system have been extensively reviewed in the past years (Clandinin and Zipursky, 2002; Borst, 2009; Borst et al., 2010; Sanes and Zipursky, 2010; Borst, 2014). Briefly, the fly visual system is composed of the compound eyes and the optic lobes of the brain (Figure 2A,B). The retina is formed by around 800 ommatidia, a repetitive basic unit of eight different photoreceptor neurons (R1–R8) and supporting cells. The photoreceptor neurons extend their axons into the brain optic lobes. Hence, the visual information travels from the retina to the four optic lobe ganglia: lamina, medulla, lobula and lobula plate (Figure 2C). The optic lobes process all the visual information including motion detection, shape, color, pattern identification, etc., and then they convey this information to the optic glomeruli in the central brain.

The optic lobe ganglia are organized in columns that receive the information from different spatial locations in the visual field establishing a retinotopic map. R1–R6 photoreceptors from neighboring ommatidia innervate a lamina cartridge, in which five types of lamina monopolar neurons (L1–L5) relay the visual information into a medulla column. On the other hand, R7 and R8 photoreceptors make synaptic connections directly into the medulla, carrying color information. The medulla is the larger ganglion, containing around 40,000 neurons (Hofbauer and Campos-Ortega, 1990) of about 80 different types (Ramón y Cajal and Sánchez, 1915; Fischbach and Dittrich, 1989; Morante and Desplan, 2008). The complete visual information from different pathways converges into the medulla, and then columnar neurons carry it into the lobula and lobula plate neuropils. The lobula plate processes the visual information required for motion detection (Borst, 2014), while the lobula ganglion links visual inputs with different behavioral responses (Wu et al., 2016; Sen et al., 2017; von Reyn et al., 2017). Finally, the lobula and lobula plate neurons project their axons into the central brain visual centers (optic glomeruli). Interestingly, some visual projection neurons directly connect the optic lobe with mushroom body Kenyon cells (Vogt et al., 2016).

After analyzing the complex neuronal network that carries the visual stimuli, the key question is how these structures are formed during development. From the early histological studies of *Drosophila* development we know that the optic lobe originates from the embryonic optic placode that is organized into two optic anlages: the inner (IPC) and outer (OPC) proliferation centers. The OPC generates the neurons of the outer medulla and the lamina, while the IPC produces the lobula complex (lobula and lobula plate) and the inner medulla neurons (Fischbach and Dittrich, 1989; Nassif et al., 2003; Ngo et al., 2017). In the next sections, we will review and discuss the early development of the optic lobe, giving special attention to recent findings that describe molecular mechanisms of neurogenesis in the lobula plate ganglion.

## Early Development of the Optic Lobe: From the Embryonic Optic Placode to the Larval Proliferation Centers

Around stage 11 of *Drosophila* embryonic development, a small group of 30–40 epithelial cells at the posterior procephalic region of the embryonic head forms the optic placode (also referred in the literature as the optic lobe primordium), which gives rise to the entire adult optic lobe (Hartenstein and Campos-Ortega, 1984; Green et al., 1993). This placode originates the Bolwig’s organs (the larval visual system) and the adult optic lobe (Green et al., 1993). While Bolwig’s organ cells remain in the head epidermis, the presumptive optic lobe cells invaginate during embryonic stages 12 and 13, maintaining their neuroepithelial morphology, and then attaching to the ventrolateral surface of the brain (Green et al., 1993; Figure 2D).

At the molecular level, the establishment and compartmentalization of the optic placode follow a genetic mechanism [signaling pathways and components of the retinal determination gene network (Silver and Rebay, 2005; Davis and Rebay, 2017)] that will be used repeatedly during the entire development of the visual system. The optic placode is recognized by the expression of several transcription factors. Sine oculis (So) is expressed in the entire optic placode (Cheyette et al., 1994; Daniel et al., 1999; Suzuki and Saigo, 2000) and is necessary for the presumptive optic lobe invagination and the formation of the Bolwig’s organ (Cheyette et al., 1994). Other transcription factors expressed in the optic placode are Lethal of scute (L’sc) (Younossi-Hartenstein et al., 1996), Tailless (Tll) and Optomotor-blind (Omb) (Poeck et al., 1993; Cheyette et al., 1994; Rudolph et al., 1997; Younossi-Hartenstein et al., 1997; Daniel et al., 1999). While the specific role of L’sc remains unknown, Tll promotes the fate of the presumptive optic lobe by blocking the development of the Bolwig’s organ (Daniel et al., 1999). On the other hand, the bHLH transcription factor Atonal (Ato) is expressed in the presumptive Bolwig’s organ and acts as “*master gene*” promoting photoreceptor differentiation (Daniel et al., 1999; Suzuki and Saigo, 2000; Figure 2D). The expression of Ato in the presumptive Bolwig’s organ is regulated by the combined action of So, Eyes absent (Eya) and the Hedgehog (Hh) signaling (Suzuki and Saigo, 2000), and repressed by Tll activity (Mishra et al., 2018). The Notch receptor and its downstream targets of the Enhancer of split complex, E(spl)mβ-HLH and E(spl)mγ-HLH, are also expressed in the optic placode (Green et al., 1993; Younossi-Hartenstein et al., 1996; Mishra et al., 2018). Interestingly, the Notch signaling seems to have two main roles in optic placode patterning: initially, Notch restricts the number of cells in the presumptive Bolwig’s organ, and later it maintains the epithelial state of the optic lobe epithelium (Green et al., 1993). Thus, Notch promotes Tll expression while blocking *ato* transcription, subdividing in this way the optic placode. Later, Notch signaling also regulates the binary decision between primary and secondary photoreceptors in the Bolwig’s organ (Mishra et al., 2018). Similarly, the EGFR pathway is active in the presumptive Bolwig’s organ and promotes secondary photoreceptor differentiation (Daniel et al., 1999; Figure 2D). Thus, a precise combination of transcription factors and signaling pathways is necessary to pattern the optic placode and to determine the fate of the larval and adult visual systems.

During optic lobe invagination, the neuroepithelial cells are arrested in G2, and they resume the cell cycle only after attaching to the brain lobes. The neuroepithelial cells divide once before entering quiescence (Hakes et al., 2018). Interestingly, between stages 12 and 17, this neuroepithelium generates about 8-9 embryonic optic lobe neuroblasts (EONs) (Hakes et al., 2018). Neuroblasts are the main neural stem cell population in the *Drosophila* central nervous system, and these EONs self-renew and generate daughter cells that differentiate into neurons and glial cells. Then, EONs enter into quiescence at the end of embryogenesis (Hakes et al., 2018). After larval hatching, the optic lobe neuroepithelial cells and EONs remain in this quiescent state until the second half of the first instar larval development when proliferation begins, triggered by larval feeding (Hofbauer and Campos-Ortega, 1990; Nassif et al., 2003; Lanet et al., 2013; Hakes et al., 2018). Currently, it is unknown what types of optic lobe neurons are generated during embryogenesis, however, EONs seem to be generated from neuroepithelial cells that express OPC markers (Hakes et al., 2018), suggesting that they may contribute to the adult medulla neuropil.

Interestingly, before development of the optic lobes resumes, a clear difference between two domains is marked by the expression of the cell adhesion molecule Fasciclin 3 (Fas3). Fas3-positive neuroepithelial cells develop into the IPC, while Fas3-negative cells form the OPC (Tayler et al., 2004; Hayden et al., 2007; Apitz and Salecker, 2015; Figure 2E). At the end of the first instar larval stage, these two neuroepithelial domains are clearly separated from each other. Later, the OPC acquires a crescent shape, while IPC neuroepithelial cells form an asymmetric horseshoe (U-shape) (Hofbauer and Campos-Ortega, 1990; Nassif et al., 2003; Egger et al., 2007; Figure 2E). Proliferation increases the size of the neuroepithelium until the early-third instar larval stage, when neuroepithelial cells in the medial edge of the OPC convert into asymmetric dividing neuroblasts re-starting neurogenesis in the optic lobe (Hofbauer and Campos-Ortega, 1990; Nassif et al., 2003; Egger et al., 2007; Lanet et al., 2013).

In the past decade, many research groups have focused their work on answering key questions about the development of the OPC and the mechanism of neurogenesis in the medulla. We currently understand with great details, how the OPC neuroepithelium is compartmentalized by the coordinated action of transcription factors such as Vsx1 (Erclik et al., 2008), Optix (Gold and Brand, 2014; Erclik et al., 2017) and Retinal homeobox (Erclik et al., 2017); and by the Wingless (Wg), Decapentaplegic (Dpp), and Hedgehog (Hh) signaling pathways (Kaphingst and Kunes, 1994; Suzuki et al., 2016b; Erclik et al., 2017). These compartmentalized epithelial domains produce different types of neuronal progenies of the optic lobe (Erclik et al., 2017). Similarly, the combination of the activity of Notch (Egger et al., 2010; Ngo et al., 2010; Reddy et al., 2010; Yasugi et al., 2010; Wang et al., 2011; Weng et al., 2012; Perez-Gomez et al., 2013; Contreras et al., 2018a), JAK-STAT (Yasugi et al., 2008; Ngo et al., 2010; Wang et al., 2013; Tanaka et al., 2018) and Fat/Hippo (Reddy et al., 2010; Kawamori et al., 2011) signaling pathways promotes the amplification of OPC neuroepithelial cells. Meanwhile the transition from neuroepithelial cells into medulla neuroblasts is promoted by the activity of L’sc and the EGFR signaling pathway in a relay mechanism called “*the proneural wave”* (Yasugi et al., 2008, 2010; Kawamori et al., 2011; Sato et al., 2016; Jorg et al., 2019). In addition to these mechanisms, medulla neuroblasts are also temporally patterned by the sequential expression of a cascade of transcription factors: Hth, Klu, Ey, Slp1/2, D, and Tll (Li et al., 2013; Suzuki et al., 2013) or Dll, Ey, Slp1/2, and D in the posterior part of the OPC (also referred as glial precursor cells, GPCs, or the tips of the OPC) (Bertet et al., 2014; Suzuki et al., 2016b). These temporal cascades regulate the fate of the progeny generated at each step, generating different types of medulla neurons (see Figure 1C). All these mechanisms have been extensively revised in high standard reviews (Sato et al., 2013, 2018; Apitz and Salecker, 2014; Suzuki and Sato, 2014, 2017; Neriec and Desplan, 2016), therefore, we will concentrate on the new findings showing novel mechanisms that regulate neurogenesis in the IPC.

## The Inner Proliferation Center as a Novel Model for Studying the Mechanisms of Neurogenesis

The mechanisms that control neurogenesis in the optic lobe IPC were a mystery that remained unresolved until very recently. Although it has been well characterized at the histological level, the molecular programs that control IPC neuroepithelial patterning or the temporal origin of the lobula complex neurons were unknown.

**FIGURE 3:**
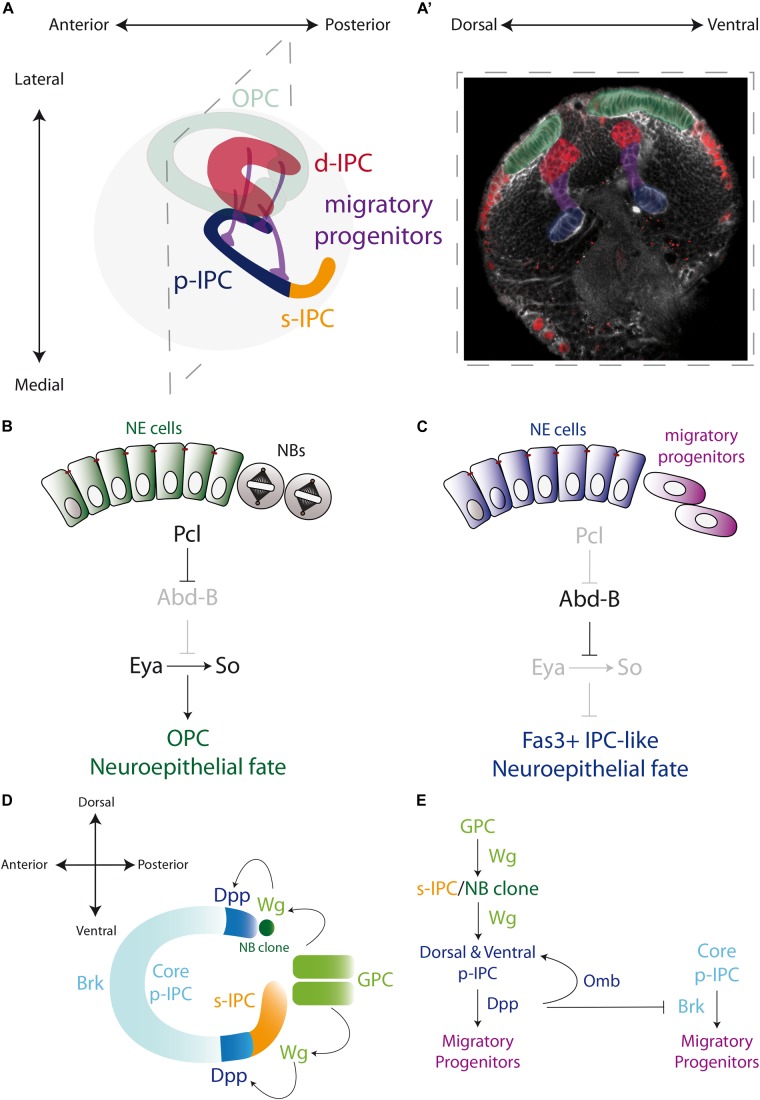
Specification of the larval optic lobe. **(A)** A schematic representation of the larval optic lobe. The optic lobe is organized in the outer proliferation center (OPC in green), and the inner proliferation center that is subdivided into four populations: surface-IPC (orange), proximal-IPC (blue), distal-IPC (red), and migratory progenitors (purple). **(A’)** A confocal optical section of the larval brain stained for E-Cadherin (gray) and Deadpan as a neuroblast marker (nuclear red) is pseudo colored to highlight the OPC (green), d-IPC (red), p-IPC (blue), and migratory progenitors (purple). **(B,C)** Specification of the OPC and IPC neuroepithelial fates by the action of Polycomblike and Abdominal-B proteins. **(D,E)** Genetic determination of the regions of the IPC neuroepithelium. Wg from the glial precursors cells (GPC) of the OPC activates the expression of Wg in the s-IPC that patterns the ventral p-IPC by expression of Dpp. Similarly, a group of OPC neuroblasts secretes Wg and induces Dpp expression in the dorsal p-IPC. In both, dorsal and ventral domains of the p-IPC, Brk expression is blocked, sharping the boundaries with the core of the IPC. Check Table 2 for a list of abbreviations.

In Apitz and Salecker (2015) established that the IPC of the third instar larva could be defined by four distinct domains according to their distinctive morphology. The *proximal IPC* (p-IPC) located at the boundary with the central brain, the *surface IPC* (s-IPC) as the superficial ventral shank of epithelial cells, and four main streams of elongated *migratory progenitor cells* connecting the p-IPC to the fourth domain, the *distal IPC* (d-IPC). The d-IPC subdomain is a C-shaped structure at the lateral part of the optic lobe, formed by neuroblasts. These neuroblasts divide to self-renew and generate neurons of the proximal medulla (C/T neurons) and the motion detection neurons of the lobula plate (T4/T5 neurons) (Apitz and Salecker, 2015; Figure 3A,A’).

## The IPC Neuroepithelium Is Organized in Different Subdomains

While the OPC is formed by a single epithelial sheet, the neuroepithelium of the IPC is much more complex. After splitting into two epithelial domains, a precise genetic mechanism determines the commitment into OPC or IPC fate. By a mutagenesis screen, Apitz and Salecker showed that the member of the Polycomb group family of chromatin-modifying proteins, Polycomblike (Pcl), is essential for the establishment of the OPC neuroepithelial fate, repressing the IPC neuroepithelial fate that acts as the optic lobe neuroepithelium default state (Apitz and Salecker, 2016). This mechanism involves the repression of the expression of the Hox gene *Abdominal-B* (*Abd-B*) by Pcl in the OPC neuroepithelium (Figure 3B). Loss of *Pcl* or ectopic expression of Abd-B triggers the formation of Fas3-positive neuroepithelial clusters in the OPC. Therefore, in the OPC Pcl is required for silencing the *Abd-B* locus to maintain the expression of the OPC markers Eya, So and Hth (Apitz and Salecker, 2016; Figure 3B,C). Interestingly, Eya, So and Hth regulate the neurogenesis mode. When they are ectopically expressed in the p-IPC, neuroepithelial cells that should convert to migratory progenitors are transformed into neuroblasts (Apitz and Salecker, 2016), confirming that this genetic network is necessary and sufficient to prevent the default IPC neuroepithelial fate.

On the other hand, little is known about how the IPC neuroepithelium grows and amplifies to form the four domains observed at the end of larval development. Similar to the OPC neuroepithelium, the Notch receptor and its ligand Delta are expressed in the IPC neuroepithelium (Wang et al., 2011), triggering the expression of the Notch signaling target genes *E(spl)m8-HLH* (Wang et al., 2011) and *E(spl)mγ-HLH* (Apitz and Salecker, 2016). Therefore, it would not be surprising that the same mechanisms that govern OPC neuroepithelium symmetric division also control neuroepithelial amplification in the IPC.

How the IPC neuroepithelium is regionalized into the proximal and superficial portions is far from being fully understood. However, it is known that the p-IPC cells switch into migratory progenitors, which eventually mature into neuroblasts; while the s-IPC neuroepithelial cells transform directly into neuroblasts (Apitz and Salecker, 2014). It is believed that these s-IPC-derived neuroblasts originate the neurons of the adult lobula ganglion (Hofbauer and Campos-Ortega, 1990; Apitz and Salecker, 2014). The expression of *Wg* and its signaling targets *frizzled 3* and *notum* in the entire s-IPC (Apitz and Salecker, 2018), suggests that only one epithelial domain is formed. Interestingly, *Wg* expression in the s-IPC is activated by Wg, which is secreted by the GPC subdomain of the OPC during early larval development, when the two epithelia are still in close proximity (Apitz and Salecker, 2018; Figure 3D).

For its part, the p-IPC is clearly subdivided into distinct domains that generate different types of neurons. The dorsal and ventral p-IPC subdomains express Dpp that in turn induces the expression of Omb and represses the expression of Brinker (Brk, a negative regulator of the Dpp pathway), restricting its expression to the central part of the p-IPC (the core subdomain) (Apitz and Salecker, 2015; Pinto-Teixeira et al., 2018; Figure 3D,E). In an extraordinary mechanism, Wg secreted from the s-IPC activates Dpp expression in the ventral p-IPC, revealing a precisely interconnected spatial patterning in the IPC neuroepithelium. Similarly, a group of OPC neuroblasts secretes the Wg ligand to activate Dpp expression in the dorsal p-IPC domain, maintaining the symmetry of the p-IPC (Apitz and Salecker, 2018; Figure 3D,E). Thus, although the initial fate of the OPC and IPC are early determined, the OPC collaborates in establishing the spatial segmentation of the IPC neuroepithelium.

## Epithelial-Mesenchymal Transition Characterizes Ipc Neurogenesis

Probably, the most striking difference between the IPC and the OPC is the presence of a new type of migratory progenitors. Unlike OPC neuroepithelial cells that convert into neuroblasts at the transition zone (Egger et al., 2007; Yasugi et al., 2008), the p-IPC cells delaminate from the neuroepithelium in a mechanism that resembles epithelial-mesenchymal transition (EMT). Hence, E-cadherin levels are decreased after conversion into migratory progenitors, in a process that requires the function of the Snail family transcription factor Escargot (Esg) and, to some extent, the activity of Dpp signaling (Apitz and Salecker, 2015). Shortly after leaving the epithelium, migratory progenitors complete the Mitosis phase of the cell cycle and arrest in S phase, while they migrate to the d-IPC (Apitz and Salecker, 2015).

**FIGURE 4:**
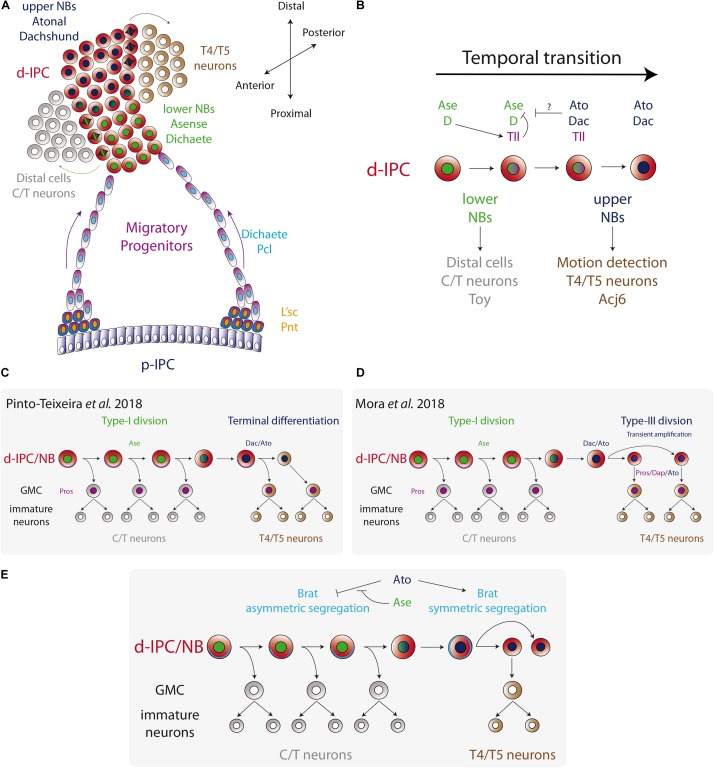
Temporal fate transition of the d-IPC neuroblasts. **(A)** p-IPC neuroepithelial cells transform into migratory progenitors that reach the distal part of the larval optic lobe and become neuroblasts. At the d-IPC, neuroblasts divide asymmetrically to generate distal cells (adult medulla C/T neurons) in that lower part of the d-IPC, while in the upper part of the d-IPC, neuroblasts generate lobula plate T4/T5 neurons. **(B)** The temporal transition between lower Ase+ neuroblasts and upper Ato+ neuroblasts is controlled by the switching factor Tll. **(C,D)** Two models for upper neuroblast mode of division. Pinto-Teixeira et al. (2018) proposed that Ato+ neuroblasts divide asymmetrically to generate a GMC and a neuroblast that undergoes terminal differentiation. On the other hand, Mora et al. (2018) postulated a new type of neuroblast symmetric division (Type-III) that transiently amplifies the pool of Ato+ neuroblasts, before entering into terminal differentiation. **(E)** A model for the regulation of Brat segregation during neuroblast mitosis. Ato blocks asymmetric segregation of Brat and switches to Type-III mode of division.

This novel migratory type of progenitors expresses weak levels of the coiled-coil adaptor protein Miranda (Mira), which controls the localization of the neuronal fate factor Prospero (Pros) in all *Drosophila* neuroblasts (Ikeshima-Kataoka et al., 1997; Shen et al., 1997; Shen et al., 1998), but with a cytoplasmic localisation (Apitz and Salecker, 2015). In addition to Mira, the stream progenitors express the Sox B-type transcription factor Dichaete (D) and Pcl (Figure 4A). When *Pcl* is mutated, the migratory progenitors prematurely upregulate the neuroblast marker Asense (Ase), showing that Pcl is required for maintaining the migratory progenitor fate (Apitz and Salecker, 2015). Similarly, ectopic expression of Abd-B resembles Pcl loss of function, triggering the premature conversion of migratory progenitors into neuroblasts (Apitz and Salecker, 2016).

How the conversion into these migratory progenitors is regulated is not well understood. L’sc and the EGFR signaling target gene Pointed (Pnt) are expressed at the inner edge of the p-IPC before the conversion into migratory progenitor (Figure 4A; Apitz and Salecker, 2015, 2016), as seen in the proneural wave of the OPC (Yasugi et al., 2008, 2010). Also, similar to OPC transition zone (Yasugi et al., 2008), *l’sc* knockdown does not correlate with major alterations in the migratory progenitors (Apitz and Salecker, 2015). However, *l’sc* knockdown associates with fewer d-IPC neuroblasts (Apitz and Salecker, 2015), suggesting a role in controlling the timing of this transition. The genetic mechanism that controls L’sc and Pnt expression and neuroepithelial cell conversion into migratory progenitors is unclear, but it would not be surprising that EGFR and Notch signaling pathways play a role in this process as they regulate the proneural wave of progenitors in the OPC (Yasugi et al., 2008, 2010). Therefore, it is plausible that the mechanism involved in the proneural wave may also control the timing of conversion of the IPC neuroepithelia. However, the outcome of this conversion is different: neuroblasts in the OPC and migratory progenitors in the IPC.

## Temporal Fate Transitions in the Ipc Neuroblasts

After leaving the p-IPC neuroepithelium, the streams of progenitors migrate to the distal part of the optic lobe (the surface of the brain) and become d-IPC neuroblasts. There, neuroblasts express the transcription factors Deadpan (Dpn) and Prospero (Pros), and switch to an asymmetric mode of division (generally referred as Type-I in the literature, see Figure 1A) to self-renew and generate a ganglion mother cell (GMC) (Apitz and Salecker, 2015). While this is classic neuroblast behavior, two competence windows define the type of progeny generated in the IPC (see Figure 1C for the temporal transition concept). In a first stage, d-IPC neuroblasts that are in close proximity to the migratory progenitors, in a region denominated the lower d-IPC (proximal part of the d-IPC), express Ase and Dichaete (D) (Figure 4A). Later, the same neuroblasts stop expressing Ase and D to turn on the expression of Dachshund (Dac) and Atonal (Ato) in the most distal part of the d-IPC (upper d-IPC) (Figure 4A; Oliva et al., 2014; Apitz and Salecker, 2015; Pinto-Teixeira et al., 2018). The switch between these two stages is controlled by the action of D and Tll (Apitz and Salecker, 2015). D activates the expression of the switching factor Tll that represses D and Ase expression. Thus, loss of *D* or *tll* prevents the conversion into Dac+/Ato+ neuroblasts, maintaining lower d-IPC identity (Figure 4B; Apitz and Salecker, 2015). Meanwhile, the knockdown of *Dac* or *Ato* does not affect the expression of each other (Apitz and Salecker, 2015).

The transition between two neural stem cell stages is the key molecular mechanism for regulating the birth order of the two main groups of IPC-derived neurons. Lower d-IPC neuroblasts position their mitotic spindles to generate distal cells next to the developing lamina. Distal cells express the transcription factors Twin of Eyeless (Toy) and later differentiate into C2, C3, T2, T2a, and T3 neurons (generically known as C/T neurons) (Apitz and Salecker, 2015). On the other hand, the upper d-IPC produces its progeny into the lobula plate cortex (the plug) to generate T4 and T5 neurons that express both Dac and the transcription factor Abnormal chemosensory jump 6 (Acj6) (Apitz and Salecker, 2015, 2018; Ngo et al., 2017; Mora et al., 2018; Figure 4A). Interestingly, simultaneous knockdown of *dac* and *ato* completely abolish T4 and T5 fates in the adult lobula plate (Apitz and Salecker, 2018), while single knockdown affects only the arborisation of T4/T5 neurons (Oliva et al., 2014; Apitz and Salecker, 2018), showing that together Dac and Ato are required for switching to the lobula plate neuronal fate.

This transition between stages not only regulates the type of progeny generated in a temporal manner, but it also switches to a different type of neuroblast division. Currently, it is known that upper d-IPC neuroblasts do not behave like Type-I neuroblasts, and how exactly these neuroblasts divide remains controversial. Recent work from the group of Claude Desplan reported that Dac+/Ato+ neuroblasts follow an asymmetric type of division, expressing weak levels of Mira and Pros that segregate into the basal cortex. However, after mitosis two cells of the same size are generated and both of them (the cell that inherited basal Mira and Pros and the Dpn+ neuroblast) acquire the GMC fate (Pinto-Teixeira et al., 2018). Therefore, upper d-IPC neuroblasts undergo terminal division to produce two differentiating progenies (Figure 4C).

On the other hand, Bassem Hassan’s group described a different mechanism for upper d-IPC neuroblast behavior. Using time-lapse imaging, Mora et al. (2018) showed that Dac+/Ato+ neuroblast division is symmetric, and both progeny cells retain nuclear Dpn expression maintaining the neuroblast fate (see Figure 1A NSC pool amplification). This novel mechanism of division, Type-III mode, allows the amplification of the upper d-IPC neuroblast pool (Mora et al., 2018). Contrary to what Pinto-Teixeira et al. (2018) reported, most of the upper d-IPC neuroblasts do not express Pros or Mira and divide symmetrically (Mora et al., 2018). However, around 15% of upper d-IPC neuroblasts express cortical Pros and Mira, which segregate into the basal cortex during mitosis, following a differentiative symmetric division as described by Mora et al. (2018); Pinto-Teixeira et al. (2018), Figure 4D.

The molecular mechanism that triggers the switch from asymmetric to symmetric division (or Type-I to Type-III mode of division) requires the function of Ato. During mitosis, lower d-IPC neuroblasts segregate the differentiation factors Brain tumor (Brat) and Numb into the basal cortex. However, they are symmetrically segregated in Dac+/Ato+ upper d-IPC neuroblasts (Mora et al., 2018). Loss of *ato* promotes Mira asymmetric localisation, while Ato misexpression in lower d-IPC neuroblasts enhances Brat localisation in a symmetric manner (Figure 4E). Furthermore, Ato directly activates Brat expression, promoting neuronal differentiation and preventing dedifferentiation (Mora et al., 2018). Therefore, Ato controls the switch from asymmetric to symmetric division of neuroblasts. However, in order to start neurogenesis of T4/T5 neurons, neuroblast symmetric division has to change from a transient amplification phase to a differentiation phase. Reduction in the size of neuroblasts has been associated with terminal differentiation in Type-I neuroblast linages (Homem et al., 2014). Similarly, upper d-IPC neuroblasts are 1.6 times smaller than lower neuroblasts, and this difference is regulated by the action of Ato. Upper d-IPC a*to* mutant neuroblasts divide asymmetrically and are bigger than their wild-type counterparts (Mora et al., 2018). Therefore, it is believed that Ato changes neuroblast division to an amplifying mode that progressively reduces cell size forcing the neuroblasts to undergo terminal differentiation.

Although, the work of Desplan’s group clearly states that they did not find any sign of neuroblast amplification, it is possible that they analyzed only the fraction of upper d-IPC neuroblasts that undergo terminal differentiation, missing the amplification step described by Mora et al. (2018). This is plausible if we consider that in the d-IPC there are twice the number of upper neuroblasts compared to lower neuroblasts (Mora et al., 2018), showing that this amplification stage does not massively increase the pool of neuroblasts. Future experimental evidence from independent groups may confirm (or reject) the presence of this novel Type-III mode of neuroblast division.

## Generating the Lobula Plate Neuronal Diversity of the Motion Detection System

Although neuroblast temporal stages regulate the main type of neuronal progeny from the IPC, this mechanism does not account for the entire IPC-derived neuronal diversity. While little is still known about how distal cells differentiate into their different progenies (C/T neuronal types), the determination of the different T4 and T5 neurons of the motion detection system has recently been elucidated.

**FIGURE 5:**
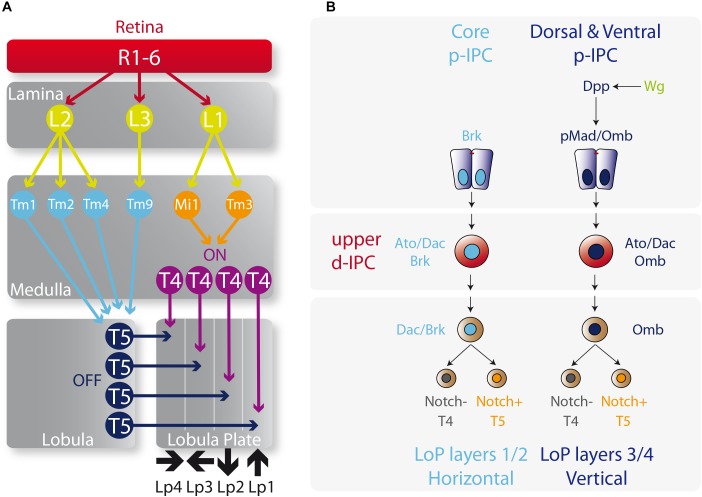
Determination of T4 and T5 neuronal fates. **(A)** Diagram of the motion visual network from the retina to T4 and T5 neurons that integrates the ON and OFF motion detection. L, lamina monopolar cells; Lp, lobula plate layer; Mi, medulla intrinsic neuron; R, photoreceptor neurons (retinula cells); Tm, transmedullary neurons. Please check the following reviews for further details on how this network functions and its role on visual detection (Borst, 2009, 2014; Borst et al., 2010; Mauss et al., 2017). **(B)** Specification of the vertical and horizontal systems. The core of the p-IPC generates the Lobula plate layers 1 and 2 of the vertical system, meanwhile the dorsal and ventral p-IPC form layers 3 and 4 of the horizontal system. The fate of T4 and T5 neurons is controlled by the repression or activation of the Notch signaling respectively.

The lobula plate receives the motion detection inputs into T4 and T5 neurons that are divided into four types (a, b, c, and d) depending on the direction of the visual movement. Hence, T4a/T5a, T4b/T5b, T4c/T5c, and T4d/T5d neurons are activated by front-to-back, back-to-front, upward, and downward motion directions, respectively (Maisak et al., 2013). Each subtype of T4 and T5 neurons projects axons into their corresponding lobula plate layers (1–4) generating two horizontal (a and b) and two vertical (c and d) layers (Figure 5A; Maisak et al., 2013).

The origin of these horizontal and vertical layers is defined by the spatial patterning of the p-IPC. Neuroepithelial cells that express Dpp (ventral and dorsal p-IPC domains) generate all the T4 and T5 neurons in the vertical layers (lobula plate layers 3 and 4), while the Brk+ population in the core of the p-IPC produces the horizontal layers (lobula plate layers 1 and 2) (Figure 5B; Apitz and Salecker, 2018; Pinto-Teixeira et al., 2018). From the Dpp neuroepithelial progeny, distal cells differentiate into C2, T2a, and T3 neurons. The Dpp target gene *omb*, is maintained active in migratory progenitors and neuroblasts, and is required for the formation of lobula plate layers 3 and 4 (Apitz and Salecker, 2018). Similarly, Dac is kept active in the T4 and T5 neurons that form the lobula plate layers 1 and 2. It seems that the default fate of the IPC is to generate layers 1 and 2 (the horizontal layers) since after Wg signaling activation, Dpp activates the transcription of *omb*, which is sufficient to repress *dac* expression and to switch the fate of IPC neural stem cells to generate layers 3 and 4 (vertical layers) (Apitz and Salecker, 2018; Figure 5B). However, the molecular mechanism that distinguishes between the directions of each layer, e.g., upward versus downward motion, remains a mystery.

The decision between the T4 and T5 fates is based on the classic binary outcome of GMC division that involves either the activation or repression of the Notch signaling pathway in one of the progenies. Thus, the progeny that activates the Notch signaling differentiates into a T5 neuron and the progeny that represses Notch signaling acquires the T4 fate (Apitz and Salecker, 2018; Pinto-Teixeira et al., 2018; Figure 5B). Moreover, the order of birth of T4 and T5 neurons determine their final location in the lobula plate cortex, and their synapse in the medulla and lobula follows the same birth order. Hence, early-born T4/T5 neurons receive visual inputs from the more posterior eye, while late-born T4/T5 neurons receive them from the anterior parts of the eye, maintaining the retinotopy across the entire visual system in a highly coordinated manner with all the optic lobe neuropils (Pinto-Teixeira et al., 2018).

## Terminal Differentiation and Connectivity Formation of the Lobula Plate Neurons

In recent years, our knowledge on the connectivity of the lobula plate network in the adult fly has increased drastically. Recent papers described in great detail the precise neuronal connectivity during the transmission of the visual information, from the eye photoreceptors to the motion sensitive T4 and T5 neurons in the lobula plate (Figure 4A; Maisak et al., 2013; Mauss et al., 2014, 2015; Serbe et al., 2016; Takemura et al., 2017). Similarly, as we described above, the current understanding of the developmental mechanisms that govern early neurogenesis as well as neuronal fate decisions during larval stages has undergone significant recent advances (Apitz and Salecker, 2015, 2016, 2018; Mora et al., 2018; Pinto-Teixeira et al., 2018). Nonetheless, we still known very little about how these neurons undergo terminal differentiation to build these well-described circuits.

**FIGURE 6:**
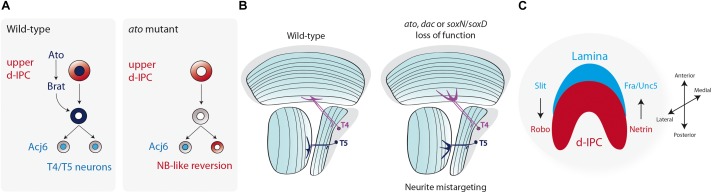
Terminal differentiation of the Lobula plate motion detection neurons. **(A)** Atonal (Ato) induces the expression of Brat and ensures the differentiation into T4 and T5 neurons. Loss of *ato* triggers the reversion of postmitotic neurons into neuroblasts. **(B)** The diagram represents a horizontal view of the adult fly optic lobe. T4 and T5 neurite targeting is regulated by the action of Ato, Dac, SoxN, and SoxD. Loss of function of these genes generates mistargeting of neurites into deeper layers of the medulla or lobula. **(C)** Schematic representation of a larval optic lobe (lateral view) showing that the compartmentalization of the optic lobe neuropils (lamina in blue, lobula plate d-IPC in red) is regulated by Slit/Robo and Netrin/Frazzled/Unc5 signaling.

Among the different differentiation steps, which include neurotransmitter synthesis and synaptic formation, dendrite and axon guidance are early events that allow the establishment of the neuronal networks. In the lobula plate neurons, not much is known about the mechanisms that regulate T4 or T5 neurite guidance. In these neurons, Ato also seems to be involved in the proper formation of T4/T5 projections. *ato* mutant larval brains show neurite misguidance phenotypes with over fasciculation defects (Oliva et al., 2014). Although it is not completely understood how Ato, which is expressed only in neuroblasts in the d-IPC, controls neurite guidance, one possibility may involve defects in acquiring the T4/T5 fate during early neurogenesis. Transcriptome analysis of *ato* mutant T4/T5 neurons revealed a reduction in the expression of genes associated with neuronal development. Moreover, ectopic neuroblasts were found mixed in the T4/T5 cortex of *ato* mutant brains, showing a dedifferentiation phenotype that requires the function of Brat (Figure 6A; Mora et al., 2018). Therefore, it is plausible that the neurite guidance phenotype observed by Oliva et al. (2014) is a consequence of early neuronal commitment defects rather than a direct effect on neurite pathfinding. Similar to *ato* mutant T4/T5 neurons, loss of *dac* function also affects neurite targeting to the medulla and lobula (Apitz and Salecker, 2018; Figure 6B). Apitz and Salecker (2018) associated this phenotype with the neurite morphology of T2/T3 neurons, which can be interpreted as a fate change from lobula plate to medulla neurons. However, a simple defect in neurite guidance could also explain this phenotype without the requirement of neuronal fate conversion.

Recently, two members of the Sox family of transcription factors have been described to participate during late development of the optic lobe. SoxNeuro (SoxN) and SoxD/Sox102F, *Drosophila* homologs of the vertebrate SoxB1 and SoxD families, respectively, control T4/T5 terminal differentiation (Contreras et al., 2018b; Schilling et al., 2019). SoxN and SoxD are only expressed in postmitotic cells in the lobula plate cortex that will differentiate into adult T4/T5 neurons, but not in neuroblasts, as is the case of Ato and Dac. Interestingly, SoxN is required for SoxD expression during differentiation (Schilling et al., 2019). Either *soxN* or *soxD* knockdown in T4/T5 neurons severely impairs neurite guidance in deeper layers of the medulla and lobula, respectively, without affecting T4/T5 neuronal fate or inducing dedifferentiation (Figure 6B; Contreras et al., 2018b; Schilling et al., 2019). Therefore, SoxN and SoxD function is only associated to the neurite guidance process, and this function is downstream T4/T5 fate determination controlled by Ato and Dac (Schilling et al., 2019).

On the other hand, the positional cues that regulate T4/T5 navigation into their correct medulla and lobula target layers are unknown; however, the axonal guidance pathways control the maintenance of the optic lobe architecture. The Netrin-Frazzled guidance system is well conserved across species (Evans, 2016), and it regulates photoreceptor and medulla neuronal guidance (Timofeev et al., 2012). The IPC derived T2 neurons, as well as the adult lobula neuropil, express the Netrin B ligand (Timofeev et al., 2012; Suzuki et al., 2018), whereas during larval development Netrin A and B are restricted to subsets of d-IPC cells (Suzuki et al., 2018). The Netrin receptor Frazzled (Fra) is broadly expressed in the adult optic lobe, but it concentrates in lamina glial cells and outer medulla neurons derived from the larval GPC (Timofeev et al., 2012; Suzuki et al., 2018). Similarly, the Netrin receptor Unc5 is expressed in GPC-derived neurons (Suzuki et al., 2018). Loss of either Netrin ligands or their receptors severely affects the compartmentalization of the larval optic lobe, generating an invasion of d-IPC neuroblasts into the OPC. Thus, Netrin signaling is required in lamina glial cells to maintain the proper boundaries between the optic lobe neuropils (Suzuki et al., 2018; Figure 6C).

The Slit-Robo family of proteins (Ypsilanti et al., 2010; Blockus and Chedotal, 2016) seems to play a similar role as the Netrin signaling in the establishment of the optic lobe architecture. The Slit ligand is highly expressed near lamina glial cells and in the medulla neuropil (Tayler et al., 2004; Fan et al., 2005; Oliva et al., 2016). *slit* mutant animals display strong defects in the compartmentalization of the lamina and lobula plate, with distal cells invading the lamina neuropil (Tayler et al., 2004). A similar, but milder phenotype is observed when *slit* is knocked down only in glial cells (Suzuki et al., 2018), suggesting that lamina glial cells signal the IPC to define the proper neuropil boundary (Figure 6C). In the IPC, distal cells express both Robo1 and Robo2 receptors that are redundantly necessary for IPC-derived distal cells to avoid entering the lamina neuropil (Tayler et al., 2004; Suzuki et al., 2016a). Similarly, the Robo3 receptor is necessary for R8 and R7 photoreceptors to avoid reaching the distal cell compartment (Pappu et al., 2011). Furthermore, Robo receptors also seem to be expressed in the lobula plate cortex at least during larval development (Tayler et al., 2004; Fan et al., 2005; Suzuki et al., 2016a). Thus, the coordinated action of the Slit-Robo and Net-Fra/Unc5 signaling maintains the architecture of the optic lobe neuropils during development (Figure 6C).

In addition to the Slit-Robo and Netrin signaling, the Ephrin-Eph system (Kania and Klein, 2016) is also a likely candidate to control lobula plate T4/T5 pathfinding. In the adult brain, the receptor Eph is highly expressed in the lobula plate neuropil and in the inner most layers of the medulla and lobula (Dearborn et al., 2002; Anzo et al., 2017). Whereas in larval stages Eph and Ephrin are expressed in the lamina, distal cells and lobula plate neurons (Dearborn et al., 2012). Dearborn et al. (2012) described that *eph* mutant larval brains show defects in the medulla neuropil, whilst *ephrin* mutants show defects in the lobula neuropil. Although all this evidence points to a role of the Netrin, Slit-Robo and Ephrin-Eph guidance pathways in the development of lobula plate neurons, their specific role on T4/T5 neurite guidance remains unknown.

## Moving From Flies Toward Mammalian Neural Development

One recurrent aspect in *Drosophila* research involves the translation of new discoveries into potential parallel mechanisms in murine models and humans. Although the anatomy of the *Drosophila* brain differs from that of mammals, there is remarkable similarity in the transcription factors and signaling pathways that control neurogenesis (Table 1). In this regard, optic lobe neurogenesis seems to be the closest invertebrate model to vertebrate neurogenesis (Brand and Livesey, 2011).

In mammals, cortical development starts from a group of neuroepithelial cells at the ventricular zone that amplifies the stem cell pool and later generates the six cortical layers. During differentiation, these neuroepithelial cells convert into radial glial cells that switch into an asymmetric self-renewing mode of division (Gotz and Huttner, 2005; Farkas and Huttner, 2008; Paridaen and Huttner, 2014). The optic lobe neuroepithelium shows several features of the mammalian cortex development, including neuroepithelial cell symmetric division (Ceron et al., 2001; Egger et al., 2007) and interkinetic nuclear migration (Rujano et al., 2013). Upon transformation into neuroblasts, asymmetric division marks the onset of neurogenesis in a similar fashion as radial glial cell division (Ceron et al., 2001; Egger et al., 2007).

During neurogenesis, cell migration and intercalation determine the final position of the neuronal progeny. Mammalian cortical development occurs in an inside-out manner in which early-born neurons form the deep layers close to the ventricular zone, while late-born neurons migrate to superficial layers of the cortex. Given the presence of a neuroepithelial pool of stem-like cells that progress into a migratory progenitor type before switching into neurogenesis (Apitz and Salecker, 2015), the *Drosophila* IPC takes the lead over the OPC in terms of similarities to mammalian neurogenesis. The IPC migratory progenitors arise from the neuroepithelium by a process that resembles epithelial to mesenchymal transition (EMT) (Apitz and Salecker, 2015). Upon neuronal fate commitment during the development of the mammalian neocortex, progenitors and neurons also migrate in a process orchestrated by EMT (Itoh et al., 2013). Similarly, the vertebrate neural crest uses EMT as a migratory strategy across the animal body before differentiating into diverse cell types, including neurons of the peripheral nervous system (Theveneau and Mayor, 2012; Munoz and Trainor, 2015). The migratory capacity of progenitors and neural stem cells is also observed in the adult mammalian brain. After a stroke, neural stem cells leave the subventricular zone to differentiate into astrocytes and repair the cortical injury (Faiz et al., 2015); whereas during homeostasis, neural precursors at the subventricular zone migrate and integrate into the olfactory bulb (Leong and Turnley, 2011; Capilla-Gonzalez et al., 2015). Thus, the fly IPC has the potential to become a novel and simple model for understanding migration during neurogenesis.

The migratory strategy in the *Drosophila* IPC involves the movement of undifferentiated progenitors to the surface of the larval brain, in contrast to the migration of postmitotic neurons in the mammalian cerebral cortex. This difference is probably due to a coordinated strategy between OPC and IPC-derived neurons. Because the OPC neuroepithelial cells are on the surface of the brain, early-born medulla neurons are pushed inside the optic lobe by late-born progeny during development. Meanwhile, IPC neuroepithelial cells, which are located inside the optic lobe, transform into migratory progenitors to reach the surface of the brain, and then neurogenesis is initiated following the same positional pattern than medulla neurons. Thus, neurons of the medulla and lobula plate are always generated in close proximity, forming the proper neuronal networks. This process is critical to maintain birth order and retinotopy across the four optic lobe neuropils and their respective layers.

Furthermore, the fact that Ato+ neuroblasts can amplify the stem cell pool (Mora et al., 2018) before their terminal differentiation (Mora et al., 2018; Pinto-Teixeira et al., 2018) adds another layer of complexity to IPC neurogenesis. The vertebrate homolog of the *Drosophila* Ato, Atonal homolog 1 (Atoh1), also seems to regulate neurogenesis. During cerebellar development, Atoh1 maintains the proliferation of granule neuron precursors (Ben-Arie et al., 1997; Flora et al., 2009; Ayrault et al., 2010). However, during spinal cord development Atoh1 plays a proneural role. Ectopic expression of Atoh1 in chick neural tube triggers a reduction in cell proliferation and premature neuronal differentiation (Nakada et al., 2004). Thus, Atoh1 is involved in the differentiation and specification of dorsal interneurons (Helms and Johnson, 1998; Gowan et al., 2001). This evidence points to a dual role of Atoh1 during vertebrate neurogenesis that resembles Ato function in the fly optic lobe development. Therefore, revealing the molecular control of optic lobe neurogenesis can give valuable information for understanding the molecular mechanisms that control mammalian central nervous system development.

## Future Directions

The development of the *Drosophila* nervous system has been an outstanding model for the comprehension of mammalian neurogenesis. For its part, the larval optic lobe is a good system for studying the different mechanisms that drive neurogenesis. In this review, we summarized the recent findings that explain the genetic control of neurogenesis of IPC-derived neurons of the optic lobe. We covered the IPC mechanisms of neurogenesis that generate neuronal diversity, which are based on the three basic principles: different modes of neural stem cell division, spatial patterning of the neurogenic region and temporal transitions of neural stem cell competence.

Despite major advances on our understanding of IPC neurogenesis, there are still several unanswered questions that need to be addressed in the future. We envision that, in the next few years, more details on the genetic mechanisms of IPC neurogenesis will be unveiled. For instance, how vertical and horizontal directions of T4/T5 neurons are specified, or which genes are either activated or repressed downstream of Notch signaling to define T5 over T4 fate and morphology. On the other hand, the exact developmental origin of the lobula neuropil needs to be revised using genetic approaches. Although early observations suggest lobula neurons are generated from the s-IPC (Hofbauer and Campos-Ortega, 1990), no clonal analysis has been performed up to date, and the mechanisms that generate lobula neuronal diversity remain unsolved. Whether different regions in the s-IPC form different lobula neurons or if a temporal pattern controlling neuronal diversity exists, needs to be assessed in the future. In the IPC domains, the dual role exhibited by Pcl seems very interesting. Pcl blocks the IPC neuroepithelial fate, but it is then necessary for the generation of migratory progenitors. How Pcl is regulated at the transcriptional level to fulfill this dual role is unknown. Finally, the specification of the different types of C/T neurons and the origin of some lobula plate neurons such as Y, Tlp (Fischbach and Dittrich, 1989) and lobula plate intrinsic neurons (LPi4-3) (Mauss et al., 2015), are not clear.

As in the past, the knowledge acquired from *Drosophila* neurogenesis, especially the development of the optic lobe, has the potential to be translated to mammalian development. We believe this information may provide a better comprehension of human embryonic and adult neurogenesis under normal and pathological conditions.

## Author Contributions

EC wrote the manuscript and designed the figures. CO and JS revised the manuscript.

## Conflict of Interest Statement

The authors declare that the research was conducted in the absence of any commercial or financial relationships that could be construed as a potential conflict of interest.
